# Support of the Laboratory in the Diagnosis of Fungal Ocular Infections

**DOI:** 10.1155/2012/643104

**Published:** 2012-03-05

**Authors:** Virginia Vanzzini Zago, Marino Alcantara Castro, Ramon Naranjo Tackman

**Affiliations:** ^1^Laboratory of Microbiology, Asociación para Evitar la Ceguera en México Hospital “Dr. Luis Sánchez-Bulnes”, Vicente Garcia Torres No. 46, 04030 Coyoacán, DF, Mexico; ^2^Cornea Department, Asociación para Evitar la Ceguera en México Hospital “Dr. Luis Sánchez-Bulnes”, Vicente Garcia Torres No. 46, 04030 Coyoacán, DF, Mexico

## Abstract

This is a retrospective, and descriptive study about the support that the laboratory of microbiology aids can provide in the diagnosis of ocular infections in patients whom were attended a tertiary-care hospital in México City in a 10-year-time period. We describe the microbiological diagnosis in palpebral mycose; in keratitis caused by *Fusarium*, *Aspergillus*, *Candida*, and melanized fungi; endophthalmitis; one *Histoplasma* scleritis and one mucormycosis. Nowadays, ocular fungal infections are more often diagnosed, because there is more clinical suspicion and there are easy laboratory confirmations. Correct diagnosis is important because an early medical treatment gives a better prognosis for visual acuity. In some cases, fungal infections are misdiagnosed and the antifungal treatment is delayed.

## 1. Introduction

Fungus is a wide group of living organisms very useful in the nature for cellulose degradation and for humans in the antibiotic synthesis and food maturation, and they have coexisted with mankind in its external and internal environment.

Even when fungal ocular infectious diseases are not frequent, actually they are more often described because there are more risky factors like corticosteroid prolonged time treatment or long-lasting broad spectrum antibiotic postsurgical treatment and intravenous drug abuse. 

Dimmer in 1913 cited by Francoise [1] published the first case of fungal panophthalmitis. In 1958, Haggerty and Zimmerman [2] described three cases of keratitis in histological studies in patients from Africa with invasion of the fungal infection to corneal epithelium, stroma, endothelium, and reaching vitreous. In Mexico, in 1950, Machado [3] reported the first ocular mycoses, and, in 1969, Gomez-Leal [4, 5] published three systemic mycoses, coccidioidomycosis, mucormycosis, and sporotrichosis with ocular and periocular involvement, with histological studies made by Sadi de Buen and Gonzalez-Ochoa [6, 7].

## 2. Material and Methods

This is a retrospective and descriptive study of patients attended in Asociacion Para Evitar la Ceguera en Mexico, Hospital “Dr. Luis Sanchez Bulnes” for their diagnosis, medical and surgical attention for fungal ocular infections, in a period of 10 years, from August 2001 to August 2011, based on clinical histories and laboratory records, including all fungal infections attended. Before starting this study, we obtained the permission from the authorities of the hospital to search the clinical history and laboratory records of each patient. 

The clinical description was made by slit lamp examination of each patient, and a sample from clinical suspected fungal infection was collected for microscopic examination and culture, with fungal identification according Jones [8]. 

In order to take the corneal sample, 2 drops of topical anesthetic (tetracaine 5 mgs/mL) were applied before obtaining smears from the corneal ulcer with heat sterilized Kimura spatula or with the sterile cotton swab (for nasal samples) and were seeded in Petri dishes with culture medium in C streaks and in slant media, for fungus and bacteria cultures. In the same way, three samples were taken for making three smears in the center of each previously cleaned slide marked with a circle made with glass pencil for microscopic observation.

For other samples like conjunctiva, orbital or sclera secretions, the samples were obtained during consultation or surgical procedures by attendant ophthalmologist and sent to the laboratory of microbiology for smears and cultures for anaerobic, aerobic bacteria, and fungus as was referred. 

For aqueous and vitreous humors, the samples were taken in the surgical room and sent to the laboratory in the same syringe, in which the samples were collected. 

Microscopic examination to identify fungi was made in each case by periodic-acid Schiff (PAS), Giemsa and Gram stains smears according to Prophet [9], and in few cases with calcofluor white and epifluorescent light microscopy. Cultures were made in a wide variety of medium; blood agar, chocolate agar in 4-5% CO_2_ ambient at 37° centigrade for bacterial growth, and for fungus Biggy agar slant for *Candida*, Sabouraud dextrose 2%, Sabouraud Emmons both with 0.01% chloramphenicol and without cycloheximide agar slant, with incubation at 27° centigrade and daily observation for a minimum of 3 weeks.

The fungal identification was made by AUXACOLOR 2 (Biorad France) absorption sugars kit for *Candida*, and cell germination forming pseudomycelium. For filamentous and melanized fungus, it was observed the morphology and pigmentation of the colony or in the medium and microcultures for its identification according to Larone [10, 11]. 

## 3. Results and Discussion

### 3.1. Fungal Blepharitis Infections

Fungal infections in the eyelids are less frequent than other fungal ocular infections, they are caused by fungus that live and have harmful effect on the skin named dermatophytes. For its hairy condition, the eyelids and eyebrows are some time involved in fungal infections in children [12] and adults. The dermatophytes described in eyelid infections are the same kind that affects the head skin (Tinea capitis), or in other skin locations (Tinea corporis), or even the intertriginous skin in hands like *Microsporum* or Trichophyton. 

**Figure 1:**
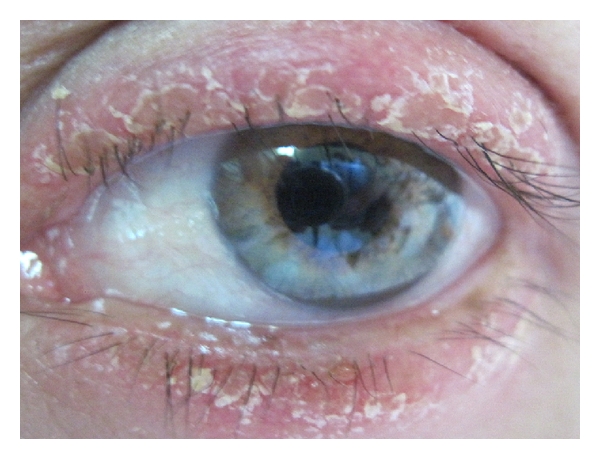
Blepharitis caused by *Trichophyton ajelloi. *

We describe a fungal blepharitis case in which at the beginning of infection it is observed like eyelid erythema, and after one week, in the same place, there were little blisters that look opened according with the patient reference, the patient had delayed fungal diagnosis and was treated with topical antibiotic ointment and steroid because it seemed to the first attendant ophthalmologist like one allergic skin reaction. After the desquamation for the breaking of the blisters, the edema remained, and then a *tinea* skin ulcer appeared, it had concentrically involvement in the skin of upper and inferior eyelid with edema, erythematic skin (Figure 1), and loss of eye lashes. 

**Figure 2:**
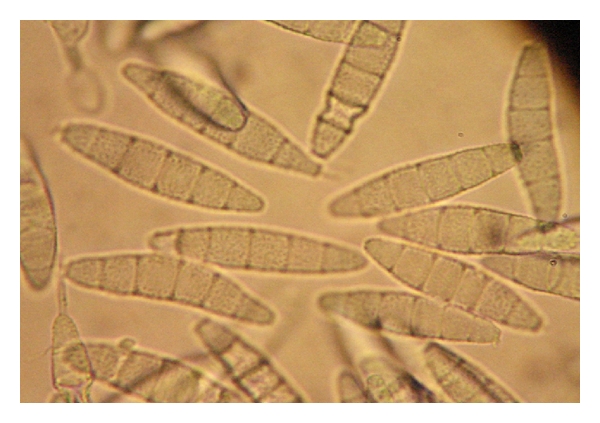
Septate conidia of *Trichophyton ajelloi* from fungal blepharitis from Figure 1 ×400.

The laboratory diagnosis was made by taking a sample of desquamating skin and staining it with periodic-acid Schiff (PAS) technique, the culture revealed after 10 days a white cottony colony that was identified as *Trichophyton ajelloi* (Figure 2).

The medical treatment was made with oral Itraconazole 100 mgs each 12 hs and topic clotrimazole ointment; the normalization of the skin and eyelid were observed after 3 weeks. 

### 3.2. Conjunctivitis by Fungal Infections

#### 3.2.1. *Candida* Conjunctivitis

Conjunctivitis caused by *Candida* has been described mainly in two life times, in the newborns and school children [13] and in the adults age [14] with the primary infection localized in oral mucosa or vagina. 

A follicular-papillary chronic conjunctivitis with no response to topic antibiotic and a slow evolution makes it suspicious to *Candida* conjunctivitis; the patients often have slow response to medical antifungal treatment; in some patients, conjunctiva membranes or pseudomembranes may be observed. 

Laboratory diagnosis was made by cultures mainly because the yeast-like cells of *Candida* often are scarce and are overlooked in the smears; the final identification was made by sugar absorption and pseudomycelium formation in a pool of human serum. *Candida albicans, C. parapsilosis,* and *C. tropicalis* had been identified as cause of conjunctivitis. 

#### 3.2.2. *Sporotrichum schenckii* Conjunctivitis

**Figure 3:**
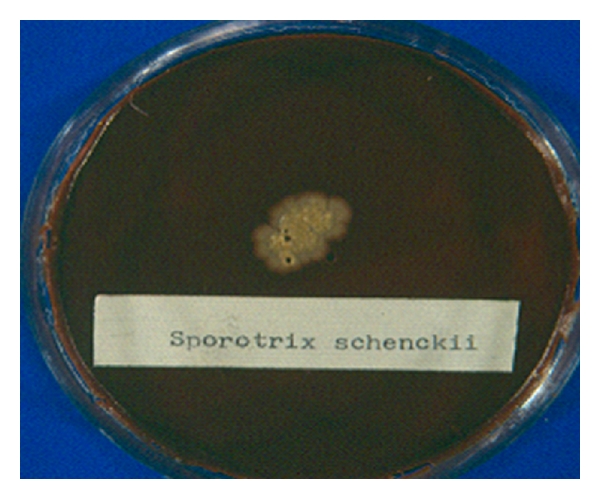
Yeast-like cells colony of *Sporothrix schenckii* isolated from conjunctiva granular tissue in, after five days of incubation in 5% CO_2_ ambient in blood agar medium.

Conjunctiva infections caused by filamentous fungus are very rare and not frequently diagnosed and published. One caused by dimorphic fungus *Sporotrichum schenckii* was reported in Japan with the diagnosis made for histological study, over bulbar conjunctiva and with a good response to topical fluconazole and oral potassium-iodine [15]. In Mexico, we attended one case culture proved, in 19-year-old female patient, the infection was located in inferior tarsal conjunctiva in right eye and external angle, with a 4 mm by 3 mm zone of granular tissue over the conjunctiva and edema around; the granular conjunctivitis started two months before being attended, preauricular enlargement of lymph node, and no systemic involvement was observed in her first consultation. Fungal culture revealed in blood agar and 5% CO_2_ white-yellow colonies formed by yeast-like cells (Figure 3), and after 10 days in Sabouraud dextrose agar grew a cottony white-gray and finally black colony that was identified by its colony morphology and microculture characteristic as *Sporothrix schenckii *[16]. 

The patient was treated with oral potassium-iodine solution, and the response was very good for conjunctiva symptoms at third day, and total cure was obtained eleven days after beginning the treatment.

### 3.3. Fungal Keratitis

Some keratitis are caused by fungus that live freely in the teleomorphic form in the environment like *Fusarium* or *Aspergillus*; this fungus in laboratory cultures shows asexual spores with meiotic or anamorphic cellular division. Cornea can be invaded for three kind of fungus; white filamentous, filamentous-melanized, and yeast-like *Candida*. Each kind of this fungus owns to groups and families in constant reclassification. Nowadays, there is an increase of new cases and reports related to soil contaminated corneal trauma, contaminated contact lens [17], inadequate disinfectant contact lens solutions, topical steroid abuse, and dry eye [18]. 

The traumatic antecedent risks are more often referred in patients in developing countries. Meanwhile, the use of contact contaminated lens was as main risk referred in developed countries, and the contaminant fungus reported was *Fusarium solani*, *Acremonium (Cephalosporium)*, *Paecilomyces*, *Candida*, *C. tropicalis*,* Curvularia, Alternaria*, and *Aspergillus *according to Wilhelmus [17].

**Figure 4:**
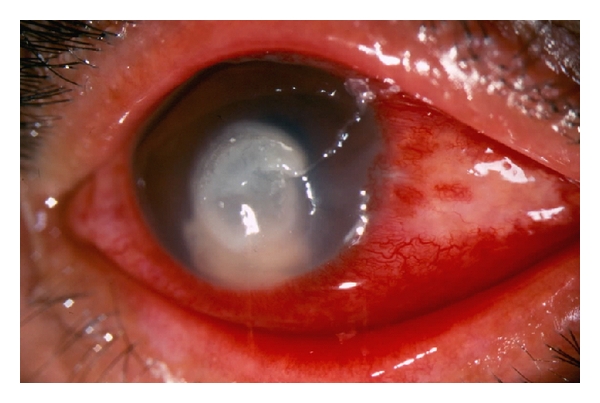
Fungal keratitis caused by *Aspergillus fumigatus*.

**Figure 5:**
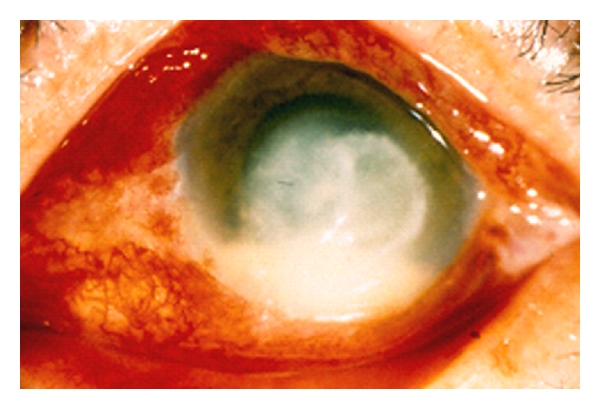
Fungal keratitis caused by melanized filamentous *Alternaria spp. *

Clinical signs and symptoms are no different from the ulcers caused by white filamentous and filamentous melanized fungus. The slow evolution, risk factors and the clinical signs are important facts for the diagnosis, the smears and cultures aid the final approach to diagnosis (Figures 4 and 5). In one serial of 219 keratitis cases studied in our center for eye care, 75.3% were male and 24.6 % were female patients with ages ranging from 8 to 94 year old and 46 median years old, 36% cases were referred previous corneal trauma, and surgical trauma in 5.4% developed postsurgery mainly caused by *Candida*.

#### 3.3.1. *Fusarium*

**Figure 6:**
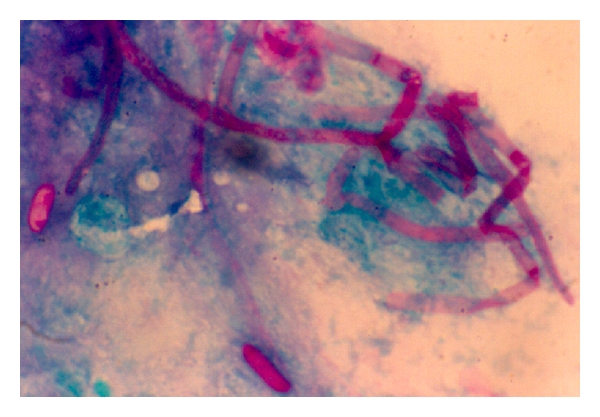
Pas stain of septate hyphae in corneal sample smears ×400.

In the corneal samples, smears with PAS stain,* Fusarium* showed septate fungal cells (hyphae) (Figure 6) indistinguishable to other septate fungus. The cultures grew in 48 to 72 hours in blood agar at 37° centigrade developing cottony white colonies. 

On Sabouraud dextrose 2% and Sabouraud Emmons mediums without cycloheximide, a white or red-yellowish color are developed at the reverse of the colony at 4 or 5 days of incubation. In the microculture technique identification, they showed banana-like macroconidia with 3-4 cells division and round to oval microconidia and were recognized according to Nelson [19]. 

In our patients' keratitis cases serial study, *Fusarium solani, F. dimerum, and F. oxysporum* were identified in 37.7%.

#### 3.3.2. *Aspergillus*

**Figure 7:**
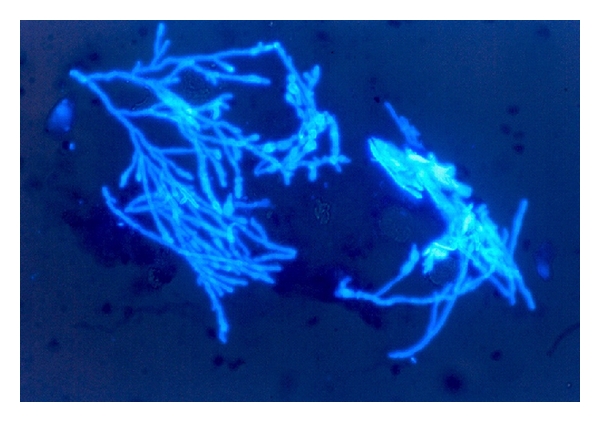
Microscopic view of fungal cells in a corneal sample smear with calcofluor and epifluorescent light ×400.

In the corneal sample smears, the hyphae showed septate cells and, in calcofluor stain technique, were indistinguishable to other moulds (Figure 7). In cultures,* Aspergillus* showed cottony white-green, or green-grey or brown-black color upon the specie colonies, with its characteristics conidiophores monoseriate or biseriate for its identification, and round to oval conidia. In our patient serial study, *Aspergillus* was isolated in 26% of keratitis cases. *Aspergillus fumigatus*, *A. nidulans*, *A. flavus*, *A. niger*, and *A. glaucus *were the most identified species.

#### 3.3.3. Filamentous Melanized Moulds

**Figure 8:**
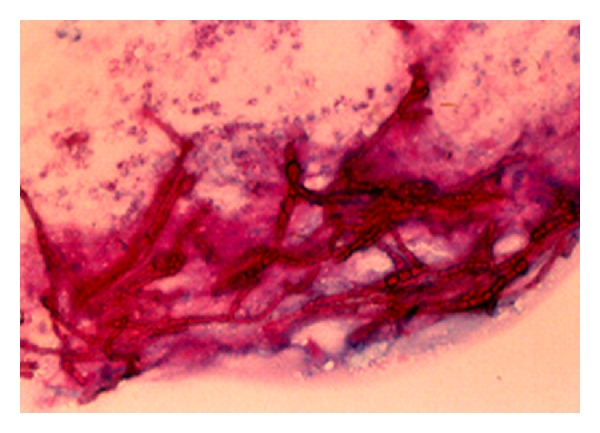
PAS stain in smear from keratitis caused by melanized fungus ×400.

Keratitis caused by filamentous melanized fungi in some cases showed brown-color over the corneal ulcer because the fungal cells are brown-color too (Figure 8). 

**Figure 9:**
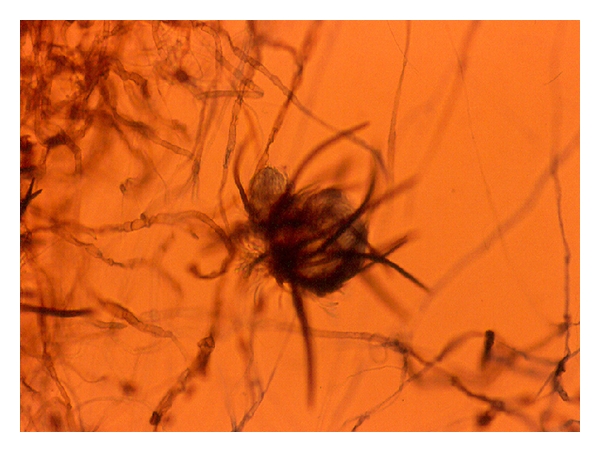
*Chaetomium globosum *isolated from a fungal keratitis in an agronomist patient ×200.

Filamentous melanized fungi were isolated from 39% of the keratitis studied [20] and were identified *Curvularia geniculata*, *Cladosporium carrionii*, *Alternaria spp*, *Phialophora spp*, *Exophiala spp*,* Wangiella spp*., *Scytalidium lignicola*, *S. dimidiatum, Phialemonium*, and, in one case, *Chaetomium globosum* (Figure 9). 

In our serial keratitis studied, the most frequent fungus involved as cause of infection was Fusarium in 37.7%, and *Aspergillus* in 26%. Traumatic antecedents were referred in 36%. In Madurai, India, Srinivasan reported in 139 fungal keratitis 47% caused by *Fusarium*, and 17% caused by *Aspergillus, *and trauma referred in 46.8%, our serial patient seemed in the relation of *Fusarium *and *Aspergillus *to the Indian author [21]. 

#### 3.3.4. *Candida spp*

**Figure 10:**
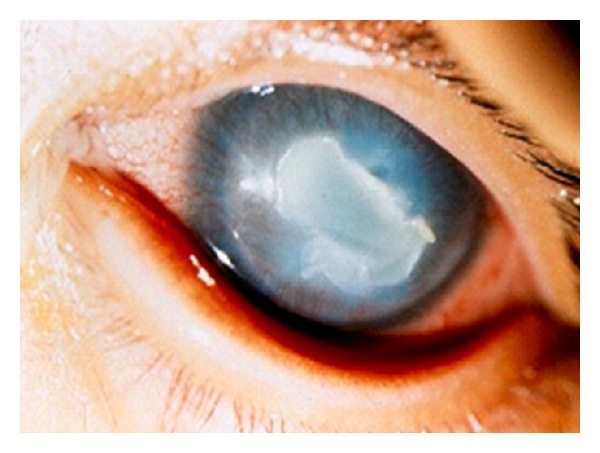
*Candida albicans* keratitis in an 8-month-old female.

**Figure 11:**
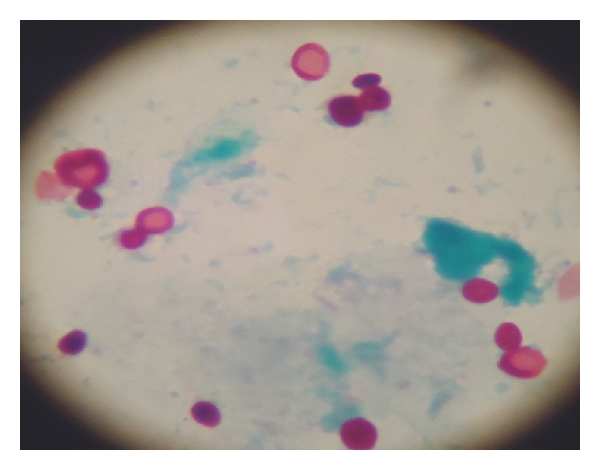
Yeast-like cells of *Candida albicans* in a smear from fungal keratitis sample stained with PAS ×1000.

*Candida* was isolated in 16% of those patients diagnosed with fungal keratitis. *Candida albicans, C. parapsilosis, C. glabrata*, and *C. tropicalis* were identified (Figures 10 and 11).

*Candida albicans* keratitis was identified in an 8-month-old female.

In temperate zones like Philadelphia Tanure, reported 24 keratitis cases in adults, and *Candida albicans* represented 45% of the fungus isolated in his serial study, and *Fusarium* only 25% [22]. 

### 3.4. Mycotic Endophthalmitis

Fungi that cause endophthalmitis reach vitreous by two ways, by trauma or surgical trauma or by endogenous way in the middle of a transient fungemia derivate from other site fungal infection, and some fungal cells reach the artery or venous retinal system.

In our serial study, we found a low frequency of fungal endophthalmitis, in 234 samples of aqueous humor and 422 samples of vitreous humor from 369 patients studied for endophthalmitis diagnosis in ten years; we obtained 189 positive cultures for bacteria or fungus, the number of fungal culture positive endophthalmitis were 17, all obtained in vitreous samples, and the percent of fungal endophthalmitis diagnosed by cultures, related to positive bacteria or fungus samples, was 8.99% (17/189). 

The antecedent risks and endophthalmitis diagnostic 2/17 (11.7%) were caused for ocular trauma and foreign body in retina, confirmed with Paecilomyces in both cases; previous surgical trauma in five cases 5/17 (29.4%) *Candida*, *Aspergillus*, *Acremonium*, and *Phoma*; in one case 1/17 (5.8%), the endophthalmitis developed after *Aspergillus niger* fungal keratitis due to cornea trauma. Endogenous endophthalmitis was 9/17 (52.9%) caused by *Candida albicans *in five* cases*; one case by *Candida dubliniensis* and one case by *Candida tropicalis*, in one case was isolated *Penicillium citrinum,* and one case with fungus cells in the smear was isolated *Scopulariopsis spp*, in the vitreous sample. In any endogenous endophthalmitis case described, the hemoculture was positive. 

**Figure 12:**
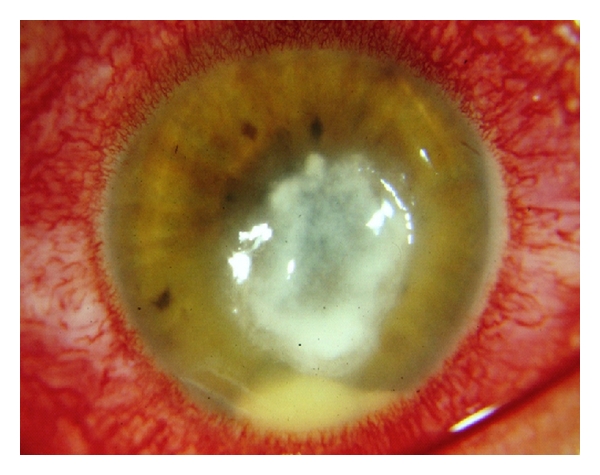
Fungal keratitis post-PRK hypopyon and central ulcer with satellite lesions.

**Figure 13:**
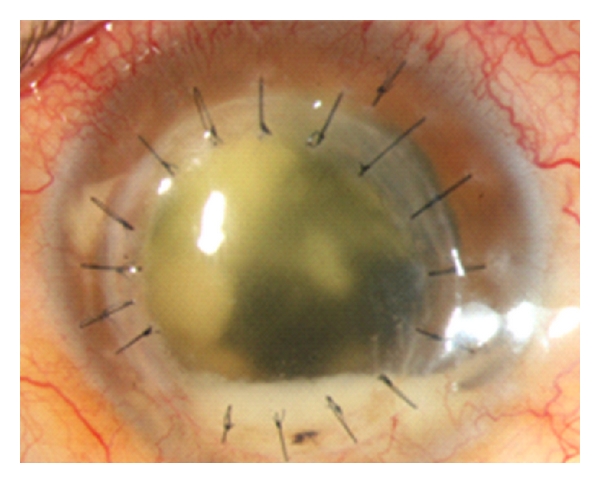
In the same case, in Figure 14, after corneal transplant for visual acuity reason, the patient developed endophthalmitis signs.

**Figure 14:**
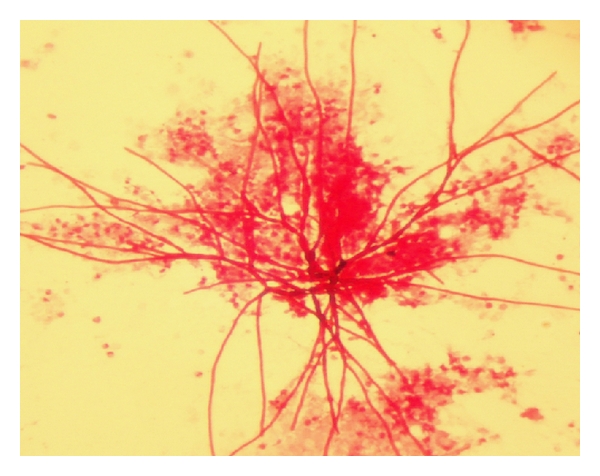
Hyphae and inflammatory cells observed in aqueous humor from case in Figure 14 ×1000.

From this series of patients, we describe one case of post- PRK (Photorefractive keratectomy) fungal keratitis. After a wide central corneal fungal keratitis, it was diagnosed (Figure 14), the patient was submitted to topical treatment with natamycin 5% suspension topical drops (Miconacina Sophia México); the therapeutic response to medical treatment was acceptable, and, for visual reasons, the patient was submitted to penetrate keratoplasty (PKP). Ten days after PKP, the patient developed a fungal endophthalmitis diagnosed by smears and cultures of aqueous and vitreous samples (Figures 12, 13, and 14).

**Figure 15:**
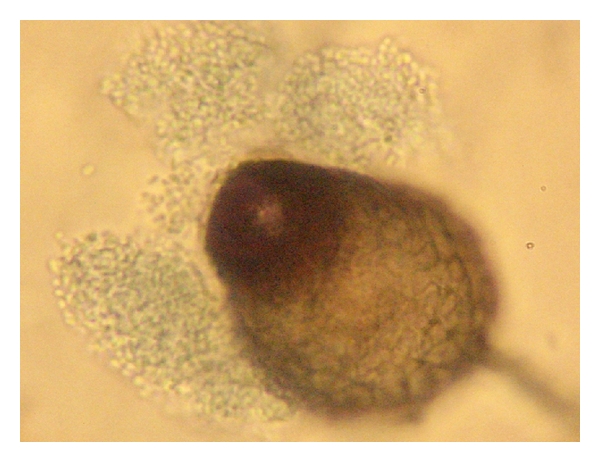
Pycnidial forms of genus Peyronellaea that identified *Phoma,* obtained in the aqueous and vitreous samples of the patient from Figure 14 ×400.

The fungus isolated was *Phoma* (Figure 15), a rare, fungus classified in genus Peyronellaea [23] associated with an endophthalmitis case described by Errera [24]. Our patient was submitted to intravitreous and oral voriconazole treatment, and the final visual acuity was diminished by retinal detachment. 

### 3.5. Fungal Sclera Infection

Sclera fungal infections are very rare events, and, in our series of cases, we described an histoplasma sclerotic infection in an immunosuppressed 47-year-old female, who was being treated for rheumatoid arthritis and dermatomyositis diagnosed 3 years ago, and treated with oral methotrexate 10 mgs weekly, azathioprine 50 mgs, and oral prednisolone 10 mgs both daily, was attended in our hospital by a red and swelled eye, with diminished ocular vision, foreign body sensation in left eye, and one sclerotic lesion in the same eye. 

At the slit lamp ocular examination, it was observed: visual acuity: left eye CF at 4 meters and corrected 1.5/10, important ecchymosis and induration in the eyelid. 

**Figure 16:**
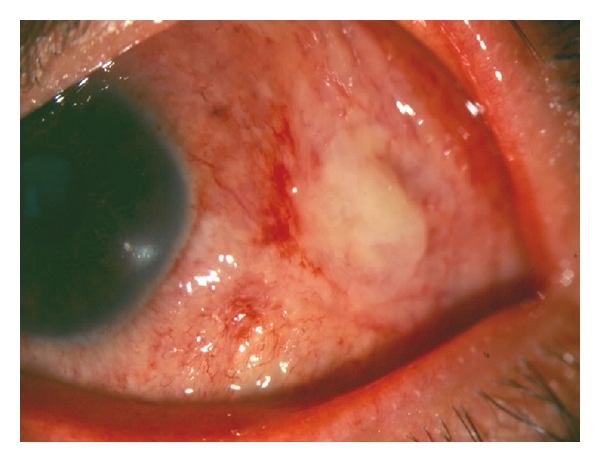
Scleritis due to *Histoplasma capsulatum *in a female immunosuppressed patient.

In conjunctiva, hyperaemia, chemosis, and ecchymosis evaluated each in ++++ and a sclerotic lesion of 4 mm located in the external angle, covered by gray-white secretion (Figure 16), were diagnosed as infectious scleritis. One sample was taken for bacteria and fungus cultures and smear as previously described. 

**Figure 17:**
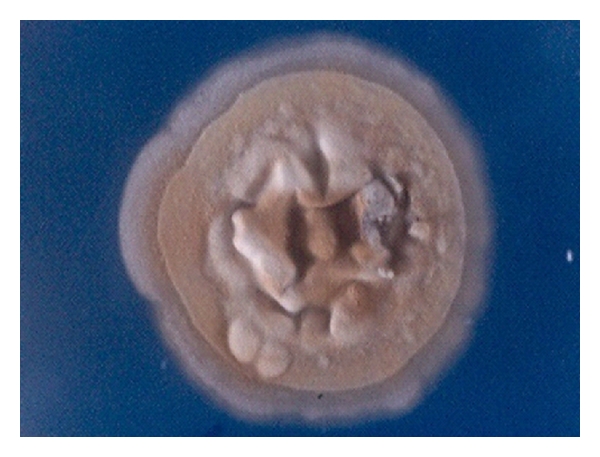
*Histoplasma capsulatum* tan colored colony after 20 days of incubation in Sabouraud-Emmons agar obtained from sclera sample culture.

**Figure 18:**
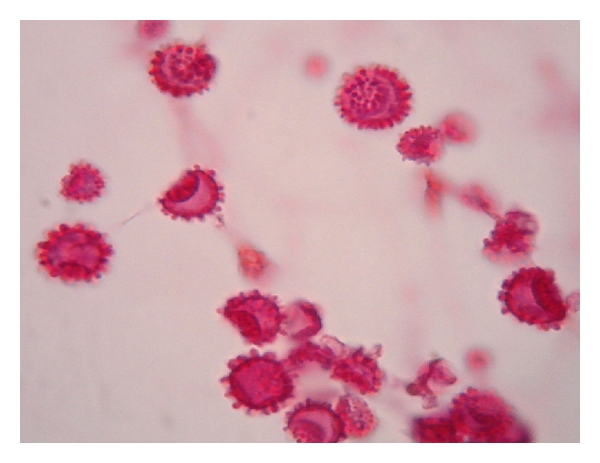
Microscopic view of microculture of *Histoplasma capsulatum* PAS stained. From scleritis case sample, described in Figure 16 ×400 magnification.

The smear with Giemsa stain showed intracellular yeast like forms in hystiocytes, and in cultures after 12 days of incubation, a cottony white colony grew in Sabouraud-Emmons media (Figure 17) and in microculture shown *Histoplasma* conidia (Figure 18), in a second sample taken from the same sclerotic ulcer, the cultures had the same microbiologic results. 

Because of the laboratory smear report, oral Itraconazole 100 mgs/day was added for treatment; prednisolone was diminished to 25 mgs by mouth each day for leucopenia detected. The ocular response to itraconazole treatment was very good, within 8 days after starting oral itraconazole. Laboratory informed the identification of fungal colonies; *Histoplasma capsulatum* var. *capsulatum* and, for the PCR identification, HC 100 protein (110 Kd) and M protein were used.

Ocular histoplasmosis culture proved is not often described; there are some endophthalmitis cases referred in immunosuppressed patients [25]. Sclerotic infections *Histoplasma capsulatum* cultures proved had not been reported before.

In Mexico, there are serologic epidemiologic studies about the infections in some risky areas [26–28]. The female patient described in histoplasmosis scleritis case lives in an endemic template zone in Morelos State, where the fructivorous bats population are high and the fruit trees are near around the living homes. We think that this together with her immunosuppression was the risk facts for the ocular infection developed. 

### 3.6. Fungal Orbital Infections

In immunosupressed patients, orbital subcutaneous mucosal infections are often caused by *Aspergillus*, *Mucor*, *Absidia*, and *Rhizopus * that are moulds that belong to the class Zygomycetes. The terms mucormycosis or zygomycoses are used to refer to the subcutaneous or deep infections like rhinoorbital, rhinocerebral, cerebral, gastrointestinal, and pulmonary mycose caused any one of these moulds. The term aspergillosis is used for pulmonary, paranasal, sinusal, or rhinoorbital fungal infrequent infection caused by diverse species of *Aspergillus *[29]. 

**Figure 19:**
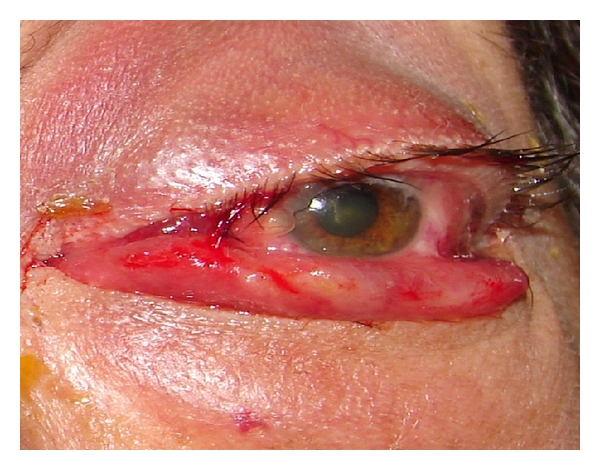
Patient showed an evident quemosis, and orbital inflammatory involvement of fungal sinusitis, diagnosed as mucormycosis.

We described the microbiologic diagnosis of one immunosuppressed patient for a kidney transplant that presented a mucormycosis that began like nasal sinusitis with orbital inflammatory involvement caused by *Rhizopus arrhizus *(formerly* Rhizopus oryzae) *(Figure 19); 30 days after the sinusitis debridement surgery and antifungal treatment, the same patient, because of work risk, was overinfected and developed a second nasal sinuses severe inflammation and a second fungal infection caused by *Aspergillus niger*.

**Figure 20:**
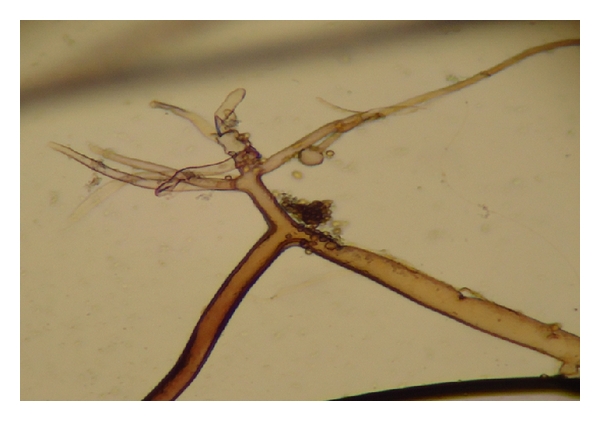
*Rhizopus arrhizus* in microculture, isolated from first sample of tissue nasal sinus debridement in patient from Figure 19 ×400 magnification.

In the first sinus debridement tissue sample, no septate hyphae was observed in PAS stain, and the culture in Sabouraud Emmons 2% without cycloheximide agar slant medium yields a fast growing fungus white-gray colony identified like *Rhizopus arrhizus* (Figure 20); in the second surgery for debridement, the samples showed a mixed fungus culture in Sabouraud Emmons without cycloheximide, and, in blood agar Petri dishes, streaked for isolation, one colony was white-gray (*Rhizopus arrhizus*) and the other was white and 3 days after turned into black on the surface, and, finally, it was identified by microculture technique like *Aspergillus niger *[30]. 

## 4. Conclusion

Ocular fungal infections fortunately are infrequent; its diagnosis, today, is easy because there are laboratory facilities for its confirmation. Some fungal keratitis are distinguishable clinically from other infectious keratitis, but clinically fungal endophthalmitis are difficult to differentiate from bacterial endophthalmitis. Fungal ocular infections treatments are difficult to manage because some antifungal drugs are not water soluble and do not penetrate in optimal concentrations into the tissues where they are useful, some like Amphotericin B and its derivatives are toxic for the delicate ocular tissues. 

For these reasons, an early clinical and laboratory diagnosis are very important for a better final visual prognosis. Preventive measures avoiding ocular trauma and adequate contact lens disinfection can diminish fungal infections in cornea. Prophylactic antifungal treatment in intensive care for patients whom are with long-lasting assisted respiratory or catheters, using an oral or intravenous dose of triazoles for fungal endophthalmitis concomitant to long-term and broad spectrum antibacterial treatment, is a good project to reduce the incidence of these intraocular infections.
